# 1-(4-Iodo-3-phenyl­isoquinolin-1-yl)pyrrolidine-2,5-dione

**DOI:** 10.1107/S1600536809042111

**Published:** 2009-10-23

**Authors:** Weijun Fu, Mei Zhu, Dongfeng Hong

**Affiliations:** a College of Chemistry and Chemical Engineering, Luoyang Normal University, Luoyang 471022, People’s Republic of China

## Abstract

In the title compound, C_19_H_13_IN_2_O_2_, the isoquinoline ring makes dihedral angles of 55.92 (3)° and 76.11 (3)° with the benzene and succinimide rings, respectively. The dihedral angle between the benzene and succinimide rings is 70.37 (3)°. In the crystal structure, the iodo atom deviates from the isoquinoline plane by 0.163 (1) Å. The crystal  studied was found to be a racemic twin with a domain ratio of 0.41 (5):0.59 (5).

## Related literature

For the synthesis of isoquinoline rings, see: Pandy *et al.* (2008 ▶). For the biological activity of isoquinolines and derivatives, see: Kletsas *et al.* (2004 ▶); Mach *et al.* (2004 ▶). For the synthesis of sterically non-hindering endocyclic ligands of the bi-isoquin­oline family and an example X-ray structure of an octa­hedral tris-chelate iron(II) complex, see: Durola *et al.* (2006 ▶). For red phospho­rescence of iridium complexes with isoquinolines and derivatives, see: Tsuboyama *et al.* (2003 ▶).
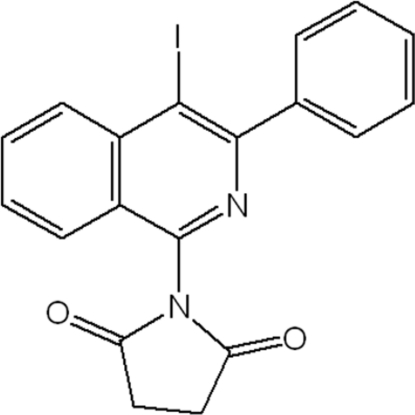


## Experimental

### 

#### Crystal data

C_19_H_13_IN_2_O_2_
*M* *_r_* = 428.21
Monoclinic, 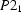

*a* = 8.874 (3) Å
*b* = 8.365 (3) Å
*c* = 11.292 (4) Å
β = 100.494 (3)°
*V* = 824.1 (4) Å^3^
*Z* = 2
Mo *K*α radiation
μ = 1.96 mm^−1^
*T* = 294 K
0.39 × 0.32 × 0.21 mm

#### Data collection

Bruker APEXII CCD diffractometer
Absorption correction: multi-scan (*SADABS*; Sheldrick, 1996 ▶) *T*_min_ = 0.516, *T*_max_ = 0.684
5068 measured reflections
2880 independent reflections
2722 reflections with *I* > 2σ(*I*)
*R*_int_ = 0.021

#### Refinement

*R*[*F* ^2^ > 2σ(*F* ^2^)] = 0.053
*wR*(*F* ^2^) = 0.154
*S* = 1.14
2880 reflections
217 parameters
1 restraint
H-atom parameters constrained
Δρ_max_ = 1.92 e Å^−3^
Δρ_min_ = −0.85 e Å^−3^
Absolute structure: Flack (1983 ▶), 1229 Friedel pairs
Flack parameter: 0.41 (5)

### 

Data collection: *APEX2* (Bruker, 2004 ▶); cell refinement: *SAINT* (Bruker, 2004 ▶); data reduction: *SAINT*; program(s) used to solve structure: *SHELXS97* (Sheldrick, 2008 ▶); program(s) used to refine structure: *SHELXL97* (Sheldrick, 2008 ▶); molecular graphics: *SHELXTL* (Sheldrick, 2008 ▶); software used to prepare material for publication: *SHELXL97* and *PLATON* (Spek, 2009 ▶).

## Supplementary Material

Crystal structure: contains datablocks global, I. DOI: 10.1107/S1600536809042111/si2208sup1.cif

Structure factors: contains datablocks I. DOI: 10.1107/S1600536809042111/si2208Isup2.hkl

Additional supplementary materials:  crystallographic information; 3D view; checkCIF report

## Figures and Tables

**Fig. 1.**
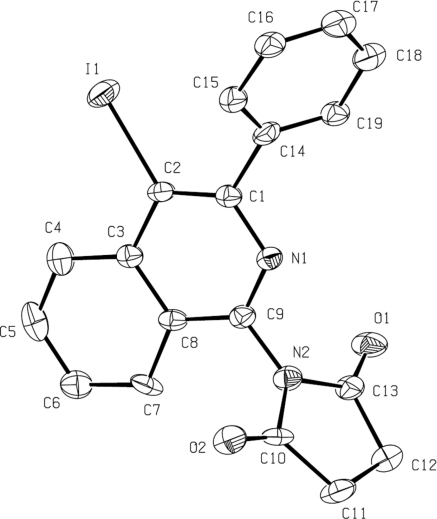
View of the molecular structure of (I) with atom numbering scheme and 30% probability displacement ellipsoids for non-hydrogen atoms.

## supplementary crystallographic information

### Comment

The isoquinoline derivatives play an important role in organic chemistry, not only as key structural units in many natural products (Kletsas *et al.*, 2004), but also as building blocks in important pharmaceuticals (Mach *et al.*, 2004). Isoquinoline species are also utilized as chiral ligands for transition metal catalysts (Durola *et al.*, 2006), and their iridium complexes are used in organic light-emitting diodes (Tsuboyama *et al.*, 2003). For these reasons, the efficient synthesis of isoquinoline ring system continues to attract the interest of synthetic chemists (Pandy *et al.*, 2008). In this context, we report the synthesis of the title compound.

The molecular structure is shown in Fig. 1. The bond lengths and angles are within normal ranges. The isoquinoline ring makes dihedral angles of 55.92 (3)° and 76.11 (3)° with the benzene and succinimide rings, respectively. The dihedral angle between the benzene and succinimide ring is 70.37 (3)°. In the crystal structure, the iodo atom deviates from the isoquinoline plane by 0.163 (1)° and the crystal is a racemic twin with a domain ratio of 0.41 (5):0.59 (5).

### Experimental

To a solution of (E)-2-(2-phenylethynyl)benzaldehyde O-acetyl oxime (0.5 mmol) in dry CH_2_Cl_2_ was added N-Iodosuccinimide (0.6 mmol). The mixture was stirred for 12 h at room temperature. After evaporation of the solvent, the residue was purified by column chromatography on silica gel to afford the title compound as a colorless solid (yield 90%). The title compound was recrystallized from CH_2_Cl_2_ at room temperature to give the desired crystals suitable for single-crystal X-ray diffraction.

### Refinement

All H atoms were positioned geometrically and treated as riding, with C—H bond lengths constrained to 0.93 Å (aromatic CH) or 0.97 Å (methylene CH_2_), and with *U*_iso_(H) = 1.2Ueq(C) or 1.5Ueq(methylene C). The number of Friedel pairs measured were 1229.

### Crystal data

**Table:**

C_19_H_13_IN_2_O_2_	*F*(000) = 420
*M**_r_* = 428.21	*D*_x_ = 1.726 Mg m^−^^3^
Monoclinic, *P*2_1_	Mo *K*α radiation, λ = 0.71073 Å
*a* = 8.874 (3) Å	Cell parameters from 3132 reflections
*b* = 8.365 (3) Å	θ = 2.3–27.9°
*c* = 11.292 (4) Å	µ = 1.96 mm^−^^1^
β = 100.494 (3)°	*T* = 294 K
*V* = 824.1 (4) Å^3^	Block, colourless
*Z* = 2	0.39 × 0.32 × 0.21 mm

### Data collection

**Table:**

Bruker APEXII CCD diffractometer	2880 independent reflections
Radiation source: fine-focus sealed tube	2722 reflections with *I* > 2σ(*I*)
graphite	*R*_int_ = 0.021
φ and ω scans	θ_max_ = 25.5°, θ_min_ = 2.3°
Absorption correction: multi-scan (*SADABS*; Sheldrick, 1996)	*h* = −10→10
*T*_min_ = 0.516, *T*_max_ = 0.684	*k* = −9→10
5068 measured reflections	*l* = −13→13

### Refinement

**Table:**

Refinement on *F*^2^	Secondary atom site location: difference Fourier map
Least-squares matrix: full	Hydrogen site location: inferred from neighbouring sites
*R*[*F*^2^ > 2σ(*F*^2^)] = 0.053	H-atom parameters constrained
*wR*(*F*^2^) = 0.154	*w* = 1/[σ^2^(*F*_o_^2^) + (0.0688*P*)^2^ + 3.9331*P*] where *P* = (*F*_o_^2^ + 2*F*_c_^2^)/3
*S* = 1.14	(Δ/σ)_max_ < 0.001
2880 reflections	Δρ_max_ = 1.92 e Å^−^^3^
217 parameters	Δρ_min_ = −0.85 e Å^−^^3^
1 restraint	Absolute structure: Flack (1983), 1229 Friedel pairs
Primary atom site location: structure-invariant direct methods	Flack parameter: 0.41 (5)

### Special details

**Table:**

Geometry. All esds (except the esd in the dihedral angle between two l.s. planes) are estimated using the full covariance matrix. The cell esds are taken into account individually in the estimation of esds in distances, angles and torsion angles; correlations between esds in cell parameters are only used when they are defined by crystal symmetry. An approximate (isotropic) treatment of cell esds is used for estimating esds involving l.s. planes.
Refinement. Refinement of F^2^ against ALL reflections. The weighted R-factor wR and goodness of fit S are based on F^2^, conventional R-factors R are based on F, with F set to zero for negative F^2^. The threshold expression of F^2^ > 2sigma(F^2^) is used only for calculating R-factors(gt) etc. and is not relevant to the choice of reflections for refinement. R-factors based on F^2^ are statistically about twice as large as those based on F, and R- factors based on ALL data will be even larger.

### Fractional atomic coordinates and isotropic or equivalent isotropic displacement parameters (Å^2^)

**Table:**

	*x*	*y*	*z*	*U*_iso_*/*U*_eq_	
I1	0.46281 (6)	0.81542 (14)	0.63285 (5)	0.0606 (3)	
C19	0.9127 (11)	0.9063 (12)	0.5388 (8)	0.042 (2)	
H19	0.9014	0.9804	0.4764	0.051*	
C17	1.0667 (9)	0.803 (3)	0.7186 (8)	0.059 (3)	
H17	1.1548	0.8082	0.7774	0.071*	
C12	0.7415 (11)	0.827 (2)	−0.0459 (8)	0.050 (2)	
H12A	0.8499	0.8453	−0.0437	0.060*	
H12B	0.6832	0.8818	−0.1150	0.060*	
C2	0.5181 (10)	0.7804 (11)	0.4622 (7)	0.039 (2)	
C14	0.8040 (9)	0.794 (2)	0.5427 (7)	0.045 (3)	
N1	0.7039 (8)	0.7695 (8)	0.3323 (6)	0.0352 (17)	
C1	0.6664 (10)	0.7796 (9)	0.4443 (7)	0.035 (2)	
C9	0.5916 (10)	0.7606 (9)	0.2384 (8)	0.0354 (19)	
C3	0.3933 (10)	0.7661 (9)	0.3598 (8)	0.0339 (19)	
C8	0.4349 (10)	0.7559 (10)	0.2450 (7)	0.0347 (18)	
C7	0.3209 (11)	0.7417 (13)	0.1405 (9)	0.048 (2)	
H7	0.3474	0.7367	0.0646	0.058*	
C5	0.1268 (12)	0.7485 (14)	0.2662 (13)	0.062 (3)	
H5	0.0240	0.7467	0.2732	0.074*	
C4	0.2361 (11)	0.7637 (11)	0.3664 (11)	0.051 (3)	
H4	0.2068	0.7728	0.4411	0.061*	
C6	0.1664 (13)	0.7355 (14)	0.1537 (10)	0.052 (2)	
H6	0.0901	0.7225	0.0861	0.062*	
C13	0.6947 (13)	0.8815 (13)	0.0688 (9)	0.045 (2)	
C15	0.8281 (12)	0.6798 (14)	0.6350 (9)	0.048 (2)	
H15	0.7552	0.6007	0.6382	0.058*	
C16	0.9609 (12)	0.6848 (15)	0.7222 (9)	0.051 (3)	
H16	0.9777	0.6080	0.7828	0.061*	
C18	1.0422 (11)	0.9091 (19)	0.6304 (10)	0.066 (4)	
H18	1.1144	0.9894	0.6293	0.079*	
C11	0.7047 (17)	0.6458 (13)	−0.0504 (10)	0.059 (3)	
H11A	0.6281	0.6213	−0.1209	0.071*	
H11B	0.7963	0.5844	−0.0544	0.071*	
O1	0.7041 (11)	1.0138 (10)	0.1076 (7)	0.061 (2)	
O2	0.6070 (9)	0.4793 (8)	0.0963 (7)	0.0532 (17)	
N2	0.6411 (10)	0.7481 (10)	0.1276 (8)	0.0408 (17)	
C10	0.6446 (12)	0.6047 (13)	0.0635 (9)	0.041 (2)	

### Atomic displacement parameters (Å^2^)

**Table:**

	*U*^11^	*U*^22^	*U*^33^	*U*^12^	*U*^13^	*U*^23^
I1	0.0499 (3)	0.0991 (6)	0.0354 (3)	0.0116 (5)	0.0148 (2)	−0.0016 (5)
C19	0.047 (5)	0.050 (5)	0.029 (5)	0.002 (4)	0.002 (4)	0.005 (4)
C17	0.033 (4)	0.098 (9)	0.040 (5)	0.021 (8)	−0.006 (3)	−0.006 (8)
C12	0.059 (5)	0.055 (6)	0.038 (4)	0.006 (7)	0.013 (4)	0.001 (7)
C2	0.042 (4)	0.049 (7)	0.025 (4)	−0.001 (4)	0.005 (3)	−0.008 (4)
C14	0.034 (4)	0.075 (9)	0.030 (4)	0.008 (5)	0.012 (3)	−0.008 (6)
N1	0.041 (4)	0.034 (4)	0.031 (3)	0.001 (3)	0.009 (3)	−0.002 (3)
C1	0.041 (4)	0.033 (6)	0.029 (4)	0.000 (3)	0.003 (3)	0.000 (3)
C9	0.048 (5)	0.027 (4)	0.033 (4)	−0.001 (3)	0.011 (4)	−0.003 (3)
C3	0.044 (4)	0.025 (5)	0.033 (4)	0.001 (3)	0.006 (3)	−0.001 (3)
C8	0.048 (5)	0.028 (4)	0.026 (4)	0.003 (3)	0.000 (3)	0.000 (3)
C7	0.044 (5)	0.055 (5)	0.038 (5)	−0.003 (4)	−0.011 (4)	−0.018 (4)
C5	0.035 (5)	0.054 (6)	0.095 (9)	−0.005 (4)	0.008 (5)	0.003 (6)
C4	0.042 (5)	0.048 (6)	0.062 (6)	0.001 (4)	0.010 (5)	0.005 (4)
C6	0.051 (6)	0.060 (6)	0.040 (5)	0.003 (5)	−0.003 (4)	0.007 (5)
C13	0.063 (6)	0.045 (6)	0.026 (4)	−0.010 (4)	0.004 (4)	0.010 (4)
C15	0.044 (5)	0.063 (6)	0.037 (5)	−0.002 (4)	0.006 (4)	0.000 (5)
C16	0.044 (5)	0.077 (7)	0.028 (5)	0.007 (5)	−0.002 (4)	0.008 (5)
C18	0.028 (5)	0.128 (11)	0.040 (6)	0.016 (5)	0.003 (4)	−0.010 (6)
C11	0.098 (9)	0.053 (7)	0.032 (5)	−0.006 (6)	0.022 (6)	−0.005 (4)
O1	0.098 (7)	0.050 (5)	0.030 (4)	−0.017 (4)	0.001 (4)	0.007 (3)
O2	0.080 (5)	0.035 (4)	0.047 (4)	0.000 (3)	0.017 (4)	0.002 (3)
N2	0.049 (4)	0.040 (4)	0.032 (4)	0.003 (3)	0.004 (3)	0.004 (3)
C10	0.049 (6)	0.049 (6)	0.022 (5)	0.006 (4)	−0.003 (4)	−0.012 (4)

### Geometric parameters (Å, °)

**Table:**

I1—C2	2.093 (8)	C3—C8	1.414 (12)
C19—C14	1.355 (17)	C8—C7	1.412 (12)
C19—C18	1.399 (14)	C7—C6	1.407 (16)
C19—H19	0.9300	C7—H7	0.9300
C17—C18	1.32 (2)	C5—C4	1.355 (16)
C17—C16	1.37 (2)	C5—C6	1.383 (17)
C17—H17	0.9300	C5—H5	0.9300
C12—C13	1.502 (14)	C4—H4	0.9300
C12—C11	1.548 (19)	C6—H6	0.9300
C12—H12A	0.9700	C13—O1	1.188 (13)
C12—H12B	0.9700	C13—N2	1.424 (12)
C2—C1	1.368 (12)	C15—C16	1.392 (13)
C2—C3	1.453 (12)	C15—H15	0.9300
C14—C15	1.399 (17)	C16—H16	0.9300
C14—C1	1.498 (11)	C18—H18	0.9300
N1—C9	1.318 (12)	C11—C10	1.519 (15)
N1—C1	1.368 (11)	C11—H11A	0.9700
C9—N2	1.402 (12)	C11—H11B	0.9700
C9—C8	1.407 (13)	O2—C10	1.181 (13)
C3—C4	1.411 (13)	N2—C10	1.404 (13)
			
C14—C19—C18	118.8 (10)	C4—C5—C6	120.7 (10)
C14—C19—H19	120.6	C4—C5—H5	119.6
C18—C19—H19	120.6	C6—C5—H5	119.6
C18—C17—C16	119.2 (9)	C5—C4—C3	121.5 (11)
C18—C17—H17	120.4	C5—C4—H4	119.3
C16—C17—H17	120.4	C3—C4—H4	119.3
C13—C12—C11	103.7 (9)	C5—C6—C7	120.7 (10)
C13—C12—H12A	111.0	C5—C6—H6	119.7
C11—C12—H12A	111.0	C7—C6—H6	119.7
C13—C12—H12B	111.0	O1—C13—N2	124.4 (9)
C11—C12—H12B	111.0	O1—C13—C12	126.1 (10)
H12A—C12—H12B	109.0	N2—C13—C12	109.4 (10)
C1—C2—C3	119.8 (8)	C16—C15—C14	120.1 (10)
C1—C2—I1	122.0 (6)	C16—C15—H15	120.0
C3—C2—I1	118.1 (6)	C14—C15—H15	120.0
C19—C14—C15	119.0 (8)	C17—C16—C15	119.9 (10)
C19—C14—C1	121.2 (10)	C17—C16—H16	120.1
C15—C14—C1	119.5 (11)	C15—C16—H16	120.1
C9—N1—C1	118.2 (7)	C17—C18—C19	123.1 (13)
C2—C1—N1	122.6 (8)	C17—C18—H18	118.5
C2—C1—C14	124.5 (8)	C19—C18—H18	118.5
N1—C1—C14	112.9 (7)	C10—C11—C12	107.3 (8)
N1—C9—N2	114.1 (8)	C10—C11—H11A	110.2
N1—C9—C8	124.6 (8)	C12—C11—H11A	110.2
N2—C9—C8	121.2 (8)	C10—C11—H11B	110.2
C4—C3—C8	118.2 (9)	C12—C11—H11B	110.2
C4—C3—C2	125.3 (9)	H11A—C11—H11B	108.5
C8—C3—C2	116.5 (8)	C9—N2—C10	124.3 (8)
C9—C8—C7	121.5 (9)	C9—N2—C13	122.9 (8)
C9—C8—C3	118.2 (8)	C10—N2—C13	112.8 (8)
C7—C8—C3	120.3 (9)	O2—C10—N2	124.3 (10)
C6—C7—C8	118.6 (10)	O2—C10—C11	129.0 (9)
C6—C7—H7	120.7	N2—C10—C11	106.7 (9)
C8—C7—H7	120.7		
			
C18—C19—C14—C15	−2.8 (16)	C6—C5—C4—C3	−0.3 (16)
C18—C19—C14—C1	−177.1 (10)	C8—C3—C4—C5	1.5 (14)
C3—C2—C1—N1	1.6 (13)	C2—C3—C4—C5	−179.4 (9)
I1—C2—C1—N1	−174.7 (6)	C4—C5—C6—C7	−1.6 (18)
C3—C2—C1—C14	−180.0 (10)	C8—C7—C6—C5	2.2 (17)
I1—C2—C1—C14	3.7 (14)	C11—C12—C13—O1	179.7 (12)
C9—N1—C1—C2	0.1 (11)	C11—C12—C13—N2	1.7 (11)
C9—N1—C1—C14	−178.5 (9)	C19—C14—C15—C16	1.0 (16)
C19—C14—C1—C2	−126.3 (10)	C1—C14—C15—C16	175.4 (10)
C15—C14—C1—C2	59.3 (15)	C18—C17—C16—C15	−1.3 (18)
C19—C14—C1—N1	52.2 (14)	C14—C15—C16—C17	1.1 (16)
C15—C14—C1—N1	−122.1 (9)	C16—C17—C18—C19	−0.6 (19)
C1—N1—C9—N2	−179.1 (7)	C14—C19—C18—C17	2.7 (17)
C1—N1—C9—C8	−2.0 (12)	C13—C12—C11—C10	−2.1 (12)
C1—C2—C3—C4	179.3 (8)	N1—C9—N2—C10	101.4 (10)
I1—C2—C3—C4	−4.2 (11)	C8—C9—N2—C10	−75.8 (12)
C1—C2—C3—C8	−1.5 (12)	N1—C9—N2—C13	−78.1 (11)
I1—C2—C3—C8	174.9 (6)	C8—C9—N2—C13	104.7 (10)
N1—C9—C8—C7	−178.2 (8)	O1—C13—N2—C9	0.8 (16)
N2—C9—C8—C7	−1.3 (13)	C12—C13—N2—C9	178.8 (8)
N1—C9—C8—C3	1.9 (12)	O1—C13—N2—C10	−178.7 (11)
N2—C9—C8—C3	178.8 (7)	C12—C13—N2—C10	−0.6 (12)
C4—C3—C8—C9	179.1 (8)	C9—N2—C10—O2	−0.3 (16)
C2—C3—C8—C9	−0.1 (11)	C13—N2—C10—O2	179.1 (10)
C4—C3—C8—C7	−0.8 (12)	C9—N2—C10—C11	179.8 (9)
C2—C3—C8—C7	−180.0 (8)	C13—N2—C10—C11	−0.8 (12)
C9—C8—C7—C6	179.1 (9)	C12—C11—C10—O2	−178.1 (11)
C3—C8—C7—C6	−1.0 (14)	C12—C11—C10—N2	1.8 (12)
